# Asthmatic Symptoms among Pupils in Relation to Winter Indoor and Outdoor Air Pollution in Schools in Taiyuan, China

**DOI:** 10.1289/ehp.10576

**Published:** 2007-10-09

**Authors:** Zhuohui Zhao, Zheng Zhang, Zhuanhua Wang, Martin Ferm, Yanling Liang, Dan Norbäck

**Affiliations:** 1 Department of Occupational and Environmental Medicine, University Hospital and Uppsala University, Uppsala, Sweden; 2 Institute of Biotechnology, Shanxi University, Taiyuan, Shanxi Province, People’s Republic of China; 3 IVL Swedish Environmental Research Institute, Gothenburg, Sweden; 4 Institute of High School Student Health Care, Taiyuan, Shanxi Province, People’s Republic of China

**Keywords:** air pollution, asthma, China, formaldehyde, indoor, nitrogen dioxide, ozone, outdoor, school, sulfur dioxide

## Abstract

**Background:**

There are few studies on associations between children’s respiratory heath and air pollution in schools in China. The industrial development and increased traffic may affect the indoor exposure to air pollutants in school environment. Moreover, there is a need to study respiratory effects of environmental tobacco smoke (ETS) and emissions from new building materials in homes in China.

**Objectives:**

We studied the associations between pupils’ asthmatic symptoms and indoor and outdoor air pollution in schools, as well as selected home exposures, in a coal-burning city in north China.

**Methods:**

A questionnaire survey was administered to pupils (11–15 years of age) in 10 schools in urban Taiyuan, collecting data on respiratory health and selected home environmental factors. Indoor and outdoor school air pollutants and climate factors were measured in winter.

**Results:**

A total of 1,993 pupils (90.2%) participated; 1.8% had cumulative asthma, 8.4% wheezing, 29.8% had daytime attacks of breathlessness. The indoor average concentrations of sulfur dioxide, nitrogen dioxide, ozone, and formaldehyde by class were 264.8, 39.4, 10.1, and 2.3 μg/m^3^, respectively. Outdoor levels were two to three times higher. Controlling for possible confounders, either wheeze or daytime or nocturnal attacks of breathlessness were positively associated with SO_2_, NO_2_, or formaldehyde. In addition, ETS and new furniture at home were risk factors for wheeze, daytime breathlessness, and respiratory infections.

**Conclusions:**

Indoor chemical air pollutants of mainly outdoor origin could be risk factors for pupils’ respiratory symptoms at school, and home exposure to ETS and chemical emissions from new furniture could affect pupils’ respiratory health.

Recent international data indicate that currently the increase of asthma and allergies in children is most pronounced in the more advanced developing countries (Asher et al. 2006). The reasons remain unclear, but this trend could be attributed to changes associated with environment and lifestyle factors during the modernization process (Douwes and Pearce 2002). Among others, the increasing level of ambient air pollution may affect children’s asthma and allergies (Schneider and Freeman 2001; Watts 2006), and indoor air pollution is another major health problem in developing countries (Bruce et al. 2000). Because China has the largest population in the world, an increase in asthma and allergies will affect a large number of individuals.

Outdoor air pollution has been a significant issue in China, especially in the coal-burning areas (Cheng et al. 2002; Mestl and Fang 2003). The respiratory effects of ambient air pollution in China have been reviewed (Aunan and Pan 2004). Coal is still the major source of energy, constituting about 75% of all energy sources (Chen et al. 2004), and consequently coal smoke, with suspended particulate matter (PM) and sulfur dioxide dominating. Furthermore, the rapidly growing number of motor vehicles accelerates emissions of other ambient air pollutants such as nitrogen dioxide and ozone (Chen et al. 2004). Ambient O_3_ levels in Shanghai, China, have been related to daily mortality in winter (Zhang et al. 2006) but not in summer, and O_3_ levels in Hong Kong have been related to asthma hospital admission in children (Lee et al. 2006).

Indoor air pollution is another important issue in China. The best known source is wood and coal burning for cooking or heating in dwellings in rural areas, which produces significant particle pollution in developing countries, including China, with pronounced impairment of respiratory health (Bruce et al. 2000). In urban areas, however, this has been widely replaced by cleaner energy sources at home, such as gas and electricity. Noticeably, chemical emissions from new building materials and furniture, such as formaldehyde, are problematic in urban areas. High indoor levels of formaldehyde have been measured in China (Cai et al. 2002), Korea (Kim and Kim 2005), and Singapore (Ooi et al. 1998). Respiratory effects of chemical emissions from new building materials, such as indoor paint, have been detected in Europe (Wieslander et al. 1997), but there is a lack of similar studies from China. Moreover, tobacco smoking is common in China, but there is not much focus on respiratory effects of environmental tobacco smoke (ETS) at home or at work (Chen et al. 1988; Qian et al. 2007; Venners et al. 2001).

Besides the dwellings, school is a particularly important indoor environment for children and adolescents. There are very few studies of school environments from mainland China (Lee and Chang 2000; Mi et al. 2006; Zhao 1991). Studies from Western countries have shown that schools can be contaminated by various indoor pollutants, such as molds, bacteria, allergens, particles, volatile organic compounds, and formaldehyde (Cooley et al. 1998; Daisey et al. 2003; Mendell and Heath 2005; Norback et al. 1990; Smedje et al. 1997). Western studies have shown that the school environment may exacerbate asthma symptoms, allergic reactions, and other respiratory symptoms (Daisey et al. 2003; Mendell and Heath 2005). Indoor air at schools in urban areas can be contaminated by ambient urban air pollution and traffic pollutants, and vicinity to busy roads may affect children’s respiratory health (Holguin et al. 2007; Janssen et al. 2003; Kim et al. 2004; Morgenstern et al. 2007).

In this study, our first aim was to examine the relationship between respiratory symptoms in junior high school students and exposure to elevated levels of air pollution in classrooms in Taiyuan city, Shanxi province, a coal-burning area in China. Another aim was to study the relationship between respiratory symptoms in the junior high school students and exposure to elevated levels of ambient air pollution from outside the schools. Our third aim was to study associations between respiratory symptoms in the students and selected exposures in the dwellings, such as ETS and new building materials and furniture—proxy variables for chemical material emissions.

## Materials and Methods

### Study design

We performed a school-based, cross-sectional study in Taiyuan, Shanxi province, China. Data on respiratory symptoms were collected by individual questionnaires as well as information on personal and home environment factors. Air pollutants including SO_2_, NO_2_, O_3_, and formaldehyde and climatic factors were measured both indoors and outdoors in classrooms and schools.

### Study locations and selection process

**Figure 1:**
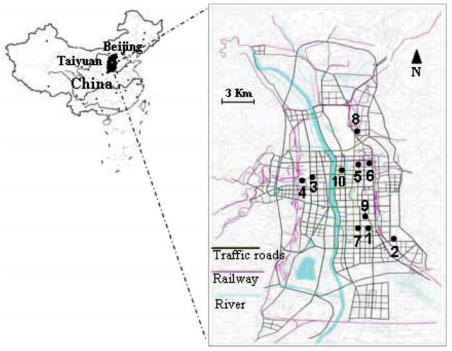
Map of the 10 selected schools in urban areas in Taiyuan, Shanxi province, China. Modified from Taiyuan Urban Planning Committee (2007).

Ten junior high schools were arbitrarily selected in December 2004 within urban areas of Taiyuan city (3 million inhabitants), situated at 500 km southwest of Beijing (Figure 1). Taiyuan is one of the most heavily polluted cities in the world, and Shanxi province is the major coal mining area, with two-thirds of China’s domestic coal production. The headmasters of 10 selected schools were contacted, and all agreed to participate.

### Study population

In each of the 10 schools, five first-year classes were arbitrary selected, in different parts and floors in the school buildings. If there were fewer than five first-year classes, all were selected. The study population consisted of 2,209 pupils (11–15 years of age) in 46 classes; 1,993 (90.2%) completed the questionnaire. There were no reports on health complaints or environmental problems from any of the schools before the investigation.

### Classroom characteristics

An inspection was performed in the 46 classrooms by the main author (Z.Z.), including measurement of room volume, floor area, number of students, and fleece factor. Fleece factor (square meters per cubic meter) was calculated as the ratio between the surface area of fabrics (square meters) and the room volume (cubic meters) (Skov et al. 1990). The schools were constructed with concrete and bricks, and none had mechanical ventilation. The floor material was bare concrete, with no paint, and the floors were cleaned by wet mopping one to three times per day by the pupils. No signs of moisture, water damage, or indoor mold growth could be observed in any of the selected classrooms, and very few classrooms had indoor plants. The mean number (± SD) of pupils per classroom was 48 ± 8 (range, 33–60), and each student occupied 1 ± 0.15 m^2^ on average (range, 0.6–1.2). The mean room volume was 193 ± 18 m^3^ (range, 161–225 m^3^). There were small amounts of curtains and upholsters in some classes; the average fleece factor was 0.03 ± 0.03 m^2^/m^3^ (range, 0–0.14).

### Air pollution measurements

Indoor levels of SO_2_, NO_2_, O_3_, and formaldehyde were measured in the selected classrooms (maximum five per school), and outdoor levels were measured at one representative location in each school by diffusion samplers. For SO_2_, NO_2_, and O_3_, samplers were obtained from IVL Swedish Environmental Research Institute Ltd. (Gothenburg, Sweden); for formaldehyde, SKC UME × 100 samplers were obtained from SKC (Eighty Four, PA, USA). The sampling time was a continuous 7-day period for each sampler. Indoor samplers were placed approximately 2 m above the floor. Outdoor samplers were placed 2.5–3.5 m above the ground, under a well-ventilated plastic cover protecting them from rain and snowfall. Thirty-four classes had indoor measurements of SO_2_, NO_2_, and O_3_, and 31 classes had measurements of formaldehyde, with measures missing in one school because of technical failures. The concentrations were analyzed by accredited laboratories specializing in analyzing the samplers, and were reported as average values across the 7-day measurement period. To evaluate how the indoor air was affected by outdoor air pollution, the ratios between indoor and outdoor concentrations were calculated.

### Climate measurements

Indoor and outdoor temperature, relative humidity (RH), and CO_2_ concentration were measured by a direct-reading instrument with in-built data logger (Q-TrakTM IAQ-monitor; TSI Inc., St Paul, MN, USA). The indoor climate measurements were performed for 1 hr with full class occupancy, during normal conditions. The outdoor measurements were performed for approximately 30 min, in parallel with the indoor measurements. Because equipment was lacking, climate measurements could only be performed in three classrooms per school at one time. We calculated the fresh air supply rate in the classrooms from the estimated equilibrium CO_2_ concentration (parts per million) by the following formula, with the equilibrium CO_2_ concentration estimated manually from the CO_2_ graphs:


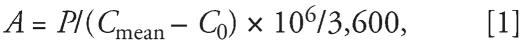


where *A* is the personal outdoor air supply rate (cubic meters per hour), *P* denotes the personal emission rate of CO_2_ in liters per hour, and *C*_mean_ and *C*_0_ denote the mean CO_2_ levels inside and outside classrooms, respectively (Norback et al. 1992). In the calculations, we assumed a personal CO_2_ emission equal to sedentary office work at sea level (18 L/hr), and used the actual outdoor CO_2_ levels from our measurements. We calculated the air exchange rate by dividing the estimated total outdoor air supply rate (cubic meters per hour) (student number × *A*) by the total volume of the classroom.

### Questionnaire

Students were given a self-administered questionnaire to collect data on their respiratory health, parental asthma or allergy, and selected factors in the home environment. Questions on respiratory health were mainly based on the International Study of Asthma and Allergy in Childhood (ISAAC) (Asher et al. 1995), the European Community Respiratory Health Survey (ECRHS) (Janson et al. 2001), and previous school studies in Sweden (Smedje et al. 1997) and in Shanghai, China (Mi et al. 2006). They included yes/no questions on cumulative asthma, doctor-diagnosed asthma, current asthma, and allergies to furry pets or pollen. Moreover, there were questions on respiratory symptoms (without using the word asthma) including wheeze or whistling in the chest, daytime or nocturnal attacks of breathlessness in the preceding 12 months, and recent respiratory infections defined as either cold, upper respiratory infection, or middle ear infection in the preceding 3 months. Finally, there were questions on parental asthma or allergy and current home environment, including recent home paintings, new floor material, and new furniture in the preceding 12 months, and ETS at home, which was classified into four categories: never smoking, or smoking one to three times per month, one to four times per week, and every day. Subjects with a lack of information on ETS were classified as a separate category. The questionnaire was translated from Swedish to Chinese and translated back to Swedish by another person. The survey was performed 1 week before the classroom inspections and measurements, distributed in the school by the class teachers, and answered at home in cooperation with the parents. The study was approved by an ethical committee of Uppsala University and performed with informed consent from pupils and parents before the study. All the personal information from questionnaire was kept confidential. All data analyses were done at the university hospital and Uppsala University, Sweden.

### Data analysis

Generally, we used multiple logistic regression model to analyze associations between response (pupils’ respiratory health on individual level ) and exposure (indoor and outdoor air pollution on class level and school level), controlling for age, sex, parental asthma or allergy, and home environmental factors (new painting, new floor material, new furniture, and ETS).

Initially, we fit a conventional logistic model by adding the continuous variables of indoor or outdoor exposure one by one (no mutual adjustment) in the model in Stata SE, version 8.0 (StataCorp., College Station, TX, USA). Second, we applied a hierarchical model for the same continuous variables, with each exposure variable separately included in the model, using MLwin 2.0 (Rasbabsh et al. 2005). Subsequently, the multivariate hierarchical regression model was fit with mutual adjustment between personal and home environmental factors and both indoor and outdoor air pollutants. In the hierarchical model, we applied a random intercept logit link–binomial model, accounting for the hierarchical structure of the data. It was estimated by iterative generalized least square, first-order marginal quasi-likelihood followed by second-order penalized quasi-likelihood. Odds ratios (OR) with 95% confidence interval (95% CIs) were applied.

Additionally, we performed sensitivity analyses, stratified by sex and parental asthma or allergy. Correlation analyses between different exposure factors were performed by a rank correlation test not requiring normal distribution (Kendall’s tau-β). In all statistical analyses, two-tailed tests and a 5% level of significance were applied.

## Results

### Questionnaire data

Prevalence of respiratory health and home environmental factors for all subjects are given in Table 1, stratified by sex, as well as prevalence in subsets of participants who were involved in the indoor measurements. Girls accounted for 49.3% of participants, and the mean age for the participants was 13 years. The prevalence of asthma or allergies was low, but respiratory symptoms were common. For daytime attacks of breathlessness, breathlessness after exercise was more common. The prevalence of demographic characteristics, home environment factors, and asthmatic symptoms was similar between total subjects and the subsets of participants. The only sex difference in symptoms was for daytime attacks of breathlessness after exercise, where girls had a higher prevalence than boys (*p* < 0.001). In home environment factors, girls reported new floor at home more often than boys (*p* < 0.001). A small percentage of pupils (11.2%) reported parental asthma or allergy. Pupils with parental asthma or allergy had a higher prevalence of cumulative incidence of asthma (*p* < 0.05), wheeze (*p* < 0.001), and daytime attacks of breathlessness (*p* < 0.001) and pollen or pet allergy (*p* < 0.05).

### Climate measurements

Climate measurements were performed in 24 classrooms. The average CO_2_ level was 2,211 ± 1,005 ppm (range, 789–4,170 ppm) with an average room temperature of 14.7 ± 2,2°C (range 11.2–18.4°C) and RH of 42 ± 10% (range 31–62%). The average air exchange rate was 2.86 ± 1.85 ac/h (range, 0.91–7.32). The average outdoor CO_2_ level was 522 ± 26 ppm (range, 480–559 ppm) with an average outdoor temperature of –1.8 ± 2,2°C (range, –5.5 to 2.6°C) and RH of 52 ± 11% (range, 30–64%).

### Indoor and outdoor SO_2_, NO_2_, O_3_, and formaldehyde

**Figure 2:**
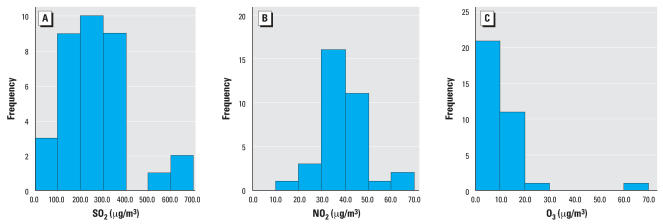
Histograms of indoor air pollutants SO_2_ (*A*), NO_2_ (*B*), and O_3_ (*C*). Data on 34 classrooms with available measurements were applied.

Descriptive data on indoor and outdoor concentrations and indoor/outdoor ratio are given in Table 2. Frequency distribution graphs of indoor SO_2_, NO_2_, and O_3_ are given in Figure 2. Indoor SO_2_ and NO_2_ were approximately normally distributed, whereas O_3_ data were more skewed. For formaldehyde, there was little variation within either indoor or outdoor concentrations, and five classrooms (from four schools) had indoor levels below the detection limit ( < 1 μg/m^3^). For SO_2_, five of 10 outdoor samples were close to saturation, and three samplers were completely saturated. In case of complete saturation, we used the saturation concentration (1,015 μg/m^3^). For SO_2_ and formaldehyde, the indoor levels were approximately 40% of the outdoor levels, for NO_2_ 78%, and for O_3_ 91%. Between indoor and outdoor levels of air pollutants, there were no significant correlations except for SO_2_ (tau-β 0.33; *p* < 0.01). Within outdoor concentrations of air pollutants, there were no significant correlations; within indoor concentrations, SO_2,_ NO_2_, and O_3_ were positively correlated, whereas there was a negative correlation between NO_2_ and formaldehyde (tau-β 0.36; *p* < 0.05). For the correlated indoor pollutants, the tau-β coefficient for SO_2_–NO_2_ was 0.74 (*p* < 0.001), SO_2_–O_3_ of 0.51 (*p* < 0.01), and NO_2_–O_3_ of 0.41 (*p* < 0.01). The indoor climate variables (temperature, RH, CO_2_, air exchange rate) were not significantly correlated with indoor pollutant concentration, except that formaldehyde had a positive correlation with room temperature (tau-β 0.41; *p* < 0.05).

### Associations between air pollution and pupils’ respiratory health

By the conventional logistic model (Table 3), results show that at higher outdoor level of formaldehyde, wheeze, and daytime attacks of breathlessness were more common. At higher indoor levels of SO_2_, wheeze and nocturnal attacks of breathlessness were more common; and at higher indoor levels of NO_2_ and formaldehyde, nocturnal breathlessness was more common. Higher levels of O_3_ suggested a marginal significance with daytime attacks of breathlessness (*p =* 0.05). However, in regard to its skewed distribution, O_3_ was additionally analyzed as categorical variable in the same models, using the 10 classrooms with lowest level as reference category, the 10 next classrooms as middle category (level 1), and the other 14 classroom with the highest O_3_ levels as the highest category (level 2). We found an association between O_3_ and daytime attacks of breathlessness: for level 1, OR = 1.65 (95% CI, 1.16–2.36), and for level 2, OR = 1.62 (95% CI, 1.15–2.29). And we found an association between O_3_ and nocturnal attacks of breathlessness: for level 1, OR = 8.69 (95% CI, 1.02–74.05), and for level 2, OR = 8.49 (95% CI, 1.01–71.06) ).

As a next step, we applied a three-level hierarchical model controlling for a cluster effect on class or school level for indoor and outdoor pollutants (Table 4). At a higher level of outdoor formaldehyde, positive associations (OR > 1) still remained with wheeze and daytime attacks of breathlessness with a slight lack of statistical significance. At higher indoor levels of SO_2_, NO_2_, and formaldehyde, nocturnal attacks of breathlessness were more common.

In the final step, we performed a three-level hierarchical model with mutual adjustment for all four indoor air pollutants, still keeping all outdoor pollutants at the school level and the same control factors as before in the individual level (Table 5). Higher levels of indoor SO_2_ were associated with more wheeze, and higher levels of indoor formaldehyde and O_3_ were associated with nocturnal attacks of breathlessness.

**Figure 3:**
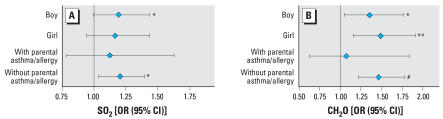
Sensitivity analysis stratified by sex and parental asthma or allergy. Two examples of sensitivity analyses were presented for associations between wheeze or whistling in the chest and indoor level of SO_2_ (*A*) and associations between daytime attacks of breathlessness and outdoor level of formaldehyde (*B*). ORs and 95% CIs were calculated by conventional logistic regression model. *p < 0.05; **p < 0.01. ^#^*p* < 0.001.

Because similar results were obtained by the hierarchic and conventional logistic regression models, we performed sensitivity analysis by conventional multiple logistic regression analysis (no mutual adjustment). Similar ORs were obtained for boys and girls, and for those with and without parental asthma/allergy. Examples are presented in Figure 3 for associations between wheeze or whistling in the chest and indoor level of SO_2_, and associations between daytime attacks of breathlessness and outdoor level of formaldehyde. The effects tended to be stronger for the subgroup without parental asthma or allergy.

### Associations between personal and home environmental factors and respiratory health

In the last three-level model, we evaluated personal factors and home environmental factors, controlled for pollutant exposure in schools. Higher age was related to more nocturnal attacks of breathlessness but fewer airway infections. Females still had more daytime attacks of breathlessness. Those with parental asthma/allergy or with new furniture at home reported more wheeze and daytime attacks of breathlessness. ETS at home was related to wheeze, daytime attacks of breathlessness, and respiratory infections, of which the latter showed a consistent associations for all three ETS categories. For comparison, we also fit conventional multiple logistic regression models, excluding controlling for school environment. Results for the above associations were similar (data not shown).

## Discussion

We found that elevated levels of air pollutants in classrooms in the heavily polluted city of Taiyuan, China, were associated with pupils’ respiratory symptoms. In addition, environmental factors at home such as ETS and emissions from new furniture seemed to exacerbate children’s respiratory symptoms. Except for formaldehyde, outdoor levels of the pollutants outside the schools were not related to symptoms.

The questionnaire survey in this study had a high response rate of 90.2%, and the questions were answered with the help of parents before the school environment measurements were started. Schools were arbitrarily selected within the urban areas of Taiyuan, and first-year classes were arbitrarily selected within the schools. There were no indications of selection bias when comparing classes participating in the questionnaire study with those included in the classroom measurements. Because the data had a three-level hierarchical structure (school, classroom, individual), we made additional analyses with three-level hierarchical models. Results were mostly consistent, with some differences in *p*-values in different models. Sensitivity analyses gave relatively similar results. Cigarette smoking is a well-established risk factor for asthma. However, four (of 1,993) students reported their own smoking. We did not control this factor in the association analysis. Thus, we have no indications of selection effects or effects of selection on a particular statistical model, but the cross-sectional study design limits the possibility of drawing conclusions based on causal relationships. Moreover, the high general air pollution level in the city may have limited the possibility of getting sufficient variation in the overall exposure to air pollutants in the study population.

The prevalence of respiratory symptoms and airway infections was high, whereas the prevalence of diagnosed asthma and allergy to furry pets or pollen was low. The discrepancy between diagnosed asthma and asthmatic symptoms, sex differences, and the validity of the symptom reporting in this school study have been discussed previously (Zhao et al. 2006). A similar low prevalence of asthma among children in Taiyuan has been reported previously in a large Chinese study (Chen 2004). Moreover, a high prevalence of respiratory symptoms and airway infections has previously been reported from other Asian school studies in Shanghai (Mi et al. 2006) and the Republic of Korea (Kim et al. 2007).

None of the schools had mechanical ventilation, and opening windows was the only way to ventilate the classrooms. This might increase the indoor level of outdoor pollutants. We could not demonstrate any correlation between air exchange rate and air pollution levels, possibly because air exchange was measured during 1 hr of normal daytime activity, whereas air pollutants were measured day and night during 1 week. Positive correlation between indoor SO_2_, NO_2_, and O_3_ could be attributed to the same major origin of these pollutants from outdoors, and the negative correlation between indoor NO_2_ and formaldehyde might indicate reactive chemistry. Further, the poor correlation between indoor and outdoor levels of pollutants indicated that the indoor exposure of these outdoor pollutants was largely determined by room-specific characteristics, such as ventilation. Because pupils spend most of their school time indoors, our study illustrates the need to measure indoor levels of air pollutants of outdoor origin in different indoor environments.

Elevated levels of indoor SO_2_ were associated with more wheeze and nocturnal attacks of breathlessness. We found very high indoor levels of SO_2_ (weekly mean, 265 μg/m^3^; range, 60–641 μg/m^3^), and outdoor levels were two to three times higher. Most samplers were over an order of magnitude higher than the World Health Organization (WHO) 24-hr standard (mean, 20 μg/m^3^ for 24-hr, and 500 μg/m^3^ for 10 min) (WHO 2005). More than half of 10 outdoor samples were close to saturation by the diffusive sampler measurement, and the outdoor levels were probably underestimated. The mean indoor/outdoor ratio for SO_2_ was 0.38 (range, 0.11–0.76), lower than indoor/outdoor ratios for NO_2_ and O_3_. This can be explained by the higher water solubility of SO_2_, which can be captured on wet surfaces such as wet concrete floor caused by frequent wet cleaning. Respiratory effects of outdoor SO_2_ have been demonstrated in other studies from China (Chen et al. 2004), but to our knowledge there are no previous studies on respiratory effects of SO_2_ exposure at school. Some studies have failed to demonstrate associations between SO_2_ and asthmatic symptoms (Garcia-Marcos et al. 1999; Goldstein and Weinstein 1986), but usually at lower exposure levels [e.g., annual mean of 75 μg/m^3^ (Garcia-Marcos et al. 1999)] than in our study. The level of outdoor particles is high in Taiyuan, and annual PM_10_ (PM with aerodynamic diameter < 10 μm) can be 252 μg/m^3^ according to the local monitoring station. In that the main source for both PM_10_ and SO_2_ is coal combustion, there should be an association between PM_10_ and SO_2_. Because we did not control for PM in our study, we could not exclude the possibility that observed health associations for SO_2_ might be attributed partly to PM exposure.

Indoor NO_2_ levels were associated with nocturnal attacks of breathlessness, both in the logistic regression and the three-level hierarchical model, but this association was not significant after mutual adjustment. The level of indoor NO_2_ was relatively high (weekly mean, 39 μg/m^3^; range, 16–62 μg/m^3^), because all schools were located in urban areas near busy roads. None of the schools had any gas heaters in the classrooms, so we did not expect any indoor sources of NO_2_. The weekly mean NO_2_ level outside the schools was 52 μg/m^3^, slightly lower than outdoor levels in a similar school study from Shanghai (weekly mean, 63 μg/m^3^), where respiratory effects of indoor NO_2_ were detected (Mi et al. 2006). The WHO air quality guideline for NO_2_ is 40 μg/m^3^ as an annual mean, and 200 μg/m^3^ for 1-hr mean (WHO 2005). It can be expected that the rapid increase of the number of cars in China will lead to a further increase of urban NO_2_ levels.

We found indications of a slight association between levels of O_3_ and respiratory symptoms in the conventional model, and these become more significant when the indoor O_3_ level was classified in three categories (low, middle, high). The weekly mean O_3_ levels were relatively low, both indoors and outdoors (10 μg/m^3^ and 12 μg/m^3^, respectively), lower than outside Shanghai schools (21 μg/m^3^) and lower than the rural background annual mean of 60 μg/m^3^ at a monitoring station (Lin An) west of Shanghai (Mi et al. 2006). It is well known that O_3_ is consumed in urban areas due to chemical reactions, for example, between O_3_ and NO to form NO_2_. Because our data are weekly means, we cannot exclude peak exposure at higher levels. Our data are not directly comparable with the WHO air quality guideline of 100 μg/m^3^ as 8-hr mean value (WHO 2005).

Surprisingly, we found associations between indoor levels of formaldehyde and wheeze and nocturnal attacks of breathlessness, and associations between outdoor formaldehyde and daytime attacks of breathlessness. In many countries, formaldehyde is considered an indoor air pollutant, but we found consistently higher levels outdoors (mean indoor/outdoor ratio, 0.38). One reason could be that formaldehyde in our study is an indicator of reactive chemistry, and possibly is associated with other stronger local irritants (Sundell and Zuber 1996; Wilkins et al. 2001). There were no temperature correction for the uptake rate of the formaldehyde diffusion samplers, but low outdoor temperature would lead to an underestimation, not an overestimation, of the true outdoor level. In Shanghai, similar outdoor levels of formaldehyde (7–9 μg/m^3^) have been measured in winter at higher outdoor temperatures (Mi et al. 2006). The reasons for our findings remain unclear, but indicate a need for more measurements of outdoor formaldehyde in Asia, and in warmer climate zones, to identify possible sources. Indoor formaldehyde levels were lower than in Shanghai schools (Mi et al. 2006) and in Swedish schools (Smedje et al. 1997). The reason may be that the schools in Taiyuan did not have new furniture, chip board, or other obvious formaldehyde sources. In Chinese dwellings with formaldehyde emissions from furniture containing chipboard, much higher formaldehyde levels (320–950 μg/m^3^) have been measured (Cai et al. 2002). Our weekly mean levels are not directly comparable with the WHO air quality guideline value of 100 μg/m^3^ as 30-min mean value (WHO 1987).

The home environment is the indoor environment where children spend most of their time. We studied selected factors in the dwellings, but had no information on type of fuel for cooking and heating, or signs of dampness or molds in the dwellings in this study, all well-known risk factors for respiratory health (Bruce et al. 2000). Data on these factors have been collected in a subsequent 2-year follow up study in the same 10 schools (unpublished data). Eighty percent used natural gas for cooking, 7% biological gas, 4% coal or coal brackets, 4% electricity, and 5% other types of fuels (including wood). Thirteen percent reported signs of dampness at home, and 3.2% reported indoor molds. This indicates that only a minority had a high exposure to particles from indoor wood or coal burning, and visible indoor molds were rare, possibly due to the cold and dry climate.

ETS exposure is also common in Chinese homes (Yang et al. 1999). After controlling for age, sex, parental asthma or allergy, and exposures at school, we found associations between ETS exposure at home and both asthmatic symptoms and respiratory infections. Significant associations with respiratory infection also presented in the subset of subjects with missing answers on ETS. This indicated there might be ETS exposure within this group (no significant associations were found for other respiratory symptoms in this group). These positive findings are in agreement with other studies on ETS (Hugg et al. 2007; Tanaka et al. 2007), but to our knowledge there are fewer data reported from China (Chen et al. 1988; Qian et al. 2007; Venners et al. 2001). Another common indoor problem in dwellings is chemical emission from new building materials such as paint, floor materials, and furniture. We found an increase of both wheeze and daytime attacks of breathlessness in homes with new furniture. This could be explained by the emission of formaldehyde or other chemicals from new furniture. To our knowledge, there are no previous international publications on associations between respiratory symptoms and new materials in Chinese dwellings.

In conclusion, indoor exposure at school to chemical air pollutants of mainly outdoor origin such as SO_2_, NO_2_, and formaldehyde was associated with asthmatic symptoms. ETS and emissions from new furniture in the dwelling could influence the prevalence of asthmatic symptoms in schoolchildren in Taiyuan city. Moreover, our study indicated a need for further measurements and epidemiologic studies on indoor and outdoor formaldehyde in China and in warmer climates. This is one of the few studies from mainland China on respiratory health effects in relation to the school environment. From a public health perspective, it is important to create a school environment that does not impair children’s respiratory health.

## Figures and Tables

**Table 1:** Demographic characteristics, home environmental factors, and asthmatic symptoms among pupils.

Characteristic	Total (*n* = 1,993)	Boys (*n* = 1,005)	Girls (*n* = 976)	With indoor air measurements^a^ (*n* = 1,480)	With indoor climate measurements^b^ (*n* = 1,056)
Age [years (mean ± SD)]	12.8 ± 0.6	12.9 ± 0.7	12.7 ± 0.6	12.8 ± 0.6	12.8 ± 0.6
Girls (%)	50.7	—	—	50.9	50.8
Parental asthma or allergy (%)	11.1	11.4	10.9	11.0	10.1
Home environmental factors (%)					
New painting	14.0	13.8	14.2	14.3	13.7
New floor	6.7	4.4	9.0	7.3	6.7
New furniture	38.3	39.2	37.4	40.7	39.6
ETS					
Never	20.1	20.7	19.5	19.9	19.2
1–3 times/month	26.5	28.2	24.9	26.5	27.9
1–4 times/week	17.1	16.9	17.3	17.5	15.6
Daily	36.4	34.2	38.3	36.1	37.3
Asthma and aasthmatic symptoms (%)					
Cumulative asthma	1.8	2.1	1.4	1.9	2.5
Doctor-diagnosed asthma	1.2	1.2	1.1	1.2	1.4
Current asthma attacks	0.4	0.5	0.3	0.5	0.7
Current asthma medication	0.5	0.4	0.6	0.5	0.5
Current airway symptoms in the preceding 12 months (%)
Wheeze or whistling in the chest	8.4	9.6	7.2	8.1	7.6
Daytime attacks of breathlessness^c^	29.8	25.8	34.0	30.5	28.9
Daytime attacks of breathlessness at rest	5.4	4.6	6.1	5.8	5.4
Daytime attacks of breathlessness after exercise	27.7	23.4	32.4	30.0	28.4
Nocturnal attacks of breathlessness	2.1	1.8	2.5	2.6	2.3
Furry pet or pollen allergy (%)	3.8	3.2	4.3	3.7	3.4
Respiratory infections in the preceding 3 months (%)	39.3	37.5	41.2	39.6	39.8

a Thirty-four of 46 classes with indoor SO_2_, NO_2_, and O_3_ measurements were included; formaldehyde was not included because three classes (from one school) had missing measurements.

b Thirty-one of 46 classes with indoor climatic measurements were included.

c Daytime attacks of breathlessness either at rest or after exercise.

**Table 2:** Indoor and outdoor air pollutants in classrooms and schools.

	No.^a^	Mean ± SD	Range
Indoor air pollutants (μg/m^3^)			
SO_2_	34	264.8 ± 139.0	60.0–641.1
NO_2_	34	39.4 ± 9.5	15.5–61.6
O_3_	34	10.1 ± 10.4	3.0–61.2
Formaldehyde	31	2.3 ± 1.1	1.0–5.0
Outdoor air pollutants (μg/m^3^)			
SO_2_	10	712.8 ± 189.3	476.0–1,015.0
NO_2_	10	52.3 ± 9.5	37.9 –65.2
O_3_	10	12.4 ± 3.3	7.1–17.5
Formaldehyde	9	5.8 ± 0.6	5.0–7.0
Indoor/outdoor ratios			
SO_2_	34	0.38 ± 0.17	0.11–0.76
NO_2_	34	0.78 ± 0.22	0.38–1.19
O_3_	34	0.91 ± 0.93	0.18–5.1
Formaldehyde	31	0.39 ± 0.18	0.14–0.83

a Number of classrooms and schools with available pollutant measurements.

**Table 3:** Conventional multiple logistic regression on asthmatic symptoms associated with indoor and outdoor air pollutants [OR (95% CI)].^a^

	Cumulative asthma	Wheeze or whistling in the chest	Daytime attacks of breathlessness	Nocturnal attacks of breathlessness	Furry pet or pollen allergy	Respiratory infection
Indoor						
SO_2_^b^	1.14 (0.85–1.54)	1.18 (1.03–1.35)*	1.07 (0.98–1.16)	1.28 (1.02–1.59)*	1.12 (0.92–1.36)	0.94 (0.86–1.02)
NO_2_^b^	1.26 (0.80–1.98)	1.12 (0.91–1.39)	1.00 (0.80–1.41)	1.45 (1.00–2.45)*	1.05 (0.77–1.42)	0.93 (0.82–1.05)
O_3_^b^	1.27 (0.95–1.71)	1.05 (0.87–1.27)	1.12 (1.00–1.26)	1.04 (0.76–1.42)	1.10 (0.87–1.39)	0.93 (0.82–1.04)
CH_2_O^c^	0.79 (0.48–1.28)	1.24 (1.03–1.48)*	1.04 (0.93–1.16)	1.40 (1.02–1.92)*	1.14 (0.89–1.46)	1.04 (0.96–1.15)
Outdoor						
SO_2_^d^	0.90 (0.73–1.11)	1.04 (0.94–1.14)	0.96 (0.90–1.02)	1.02 (0.84–1.23)	1.04 (0.91–1.19)	0.98 (0.93–1.04)
NO_2_^d^	0.71 (0.50–1.01))	1.00 (0.83–1.20)	0.97 (0.86–1.08)	1.04 (0.72–1.50)	0.98 (0.76–1.27)	0.95 (0.86–1.06)
O_3_^d^	0.65 (0.22–1.87)	0.67 (0.39–1.14)	0.85 (0.62–1.17)	0.83 (0.29–2.35)	0.66 (0.32–1.38)	0.89(0.66–1.19)
CH_2_O^e^	1.11 (0.59–2.07)	1.38 (1.03–1.85)*	1.42 (1.19–1.70)**	1.72 (0.98–3.03)	1.17 (0.78–1.74)	0.99 (0.84–1.17)

CH_2_O, formaldehyde.

a Each air pollutant variable is included in the model separately, controlling for age, sex, parental asthma or allergy, ETS at home, recent home painting, new floor and new furniture in the preceding 12 months. ORs refer to a step change of 100μg/m^3^, 10μg/m^3^, 10μg/m^3^, and 1 μg/m^3^ of SO_2_, NO_2_, O_3_, and formaldehyde, respectively. The individual exposure to air pollutants was addressed the same as the classroom level for indoor exposure and the school level for outdoor exposure, respectively.

b Applied for available data in 34 classes across 10 schools (*n* = 1,480).

c Applied for available data in 31 classes across 9 schools (*n* = 1,362).

d Applied for available data in 10 schools (*n* = 1,993).

e Applied for available data in 9 schools (*n* = 1,836).

* *p* < 0.05.

** *p* < 0.001.

For indoor O_3_ and daytime attacks of breathlessness, *p* = 0.05.

**Table 4:** Hierarchical multiple logistic regression on asthmatic symptoms associated with indoor and outdoor air pollutants [OR (95% CI)].^a^

	Cumulative asthma	Wheeze or whistling in the chest	Daytime attacks of breathlessness	Nocturnal attacks of breathlessness	Furry pet or pollen allergy	Respiratory infection
Indoor						
SO_2_	1.12 (0.71–1.76)	1.15 (0.94–1.42)	1.04 (0.90–1.20)	1.27 (1.02–1.59)*	1.12 (0.92–1.36)	0.93 (0.84–1.04)
NO_2_	1.32 (0.55–3.14)	1.04 (0.77–1.39)	0.95 (0.78–1.16)	1.45 (1.00–2.08)*	1.04 (0.76–1.43)	0.92 (0.79–1.08)
O_3_	1.21 (0.77–1.92)	0.99 (0.80–1.25)	1.08 (0.94–1.25)	1.04 (0.73–1.49)	1.10 (0.86–1.39)	0.92 (0.79–1.07)
CH_2_O	0.81 (0.49–1.33)	1.13 (0.89–1.44)	1.00 (0.84–1.19)	1.40 (1.02–1.92)*	1.15 (0.89–1.48)	1.04 (0.90–1.20)
Outdoor						
SO_2_	0.97 (0.70–1.35)	1.03 (0.82–1.29)	1.00 (0.87–1.16)	1.05 (0.83–1.32)	1.06 (0.89–1.27)	0.98 (0.89–1.08)
NO_2_	0.66 (0.37–1.18)	1.02 (0.65–1.60)	0.92 (0.60–1.22)	0.94 (0.59–1.51)	1.00 (0.71–1.40)	0.89 (0.74–1.07)
O_3_	0.50 (0.11–2.28)	0.59 (0.20–1.27)	0.64 (0.19–1.96)	0.61 (0.19–1.96)	0.70 (0.29–1.67)	0.84 (0.52–1.33)
CH_2_O	1.89 (0.83–4.32)	1.64 (0.96–2.83)	1.36 (0.99–1.86)	1.63 (0.90–2.95)	1.09 (0.68–1.75)	0.92 (0.72–1.18)

CH_2_O, formaldehyde.

a Three-level hierarchical logistic model (school–class–student) was applied for the same available data as in conventional logistic regression model (see Table 3 for available data information). Each air pollutant was included in the model separately, controlling for age, sex, parental asthma or allergy, ETS at home, recent home painting, new floor and new furniture in the preceding 12 months. ORs refer to a step change of 100 μg/m^3^, 10 μg/m^3^, 10 μg/m^3^, and 1 μg/m^3^ of SO_2_, NO_2_, O_3_, and formaldehyde, respectively.

* *p* < 0.05.

**Table 5:** Hierarchical multiple logistic model with mutual adjustment for indoor air pollutants associated with asthmatic symptoms [OR (95% CI)].^a^

	Cumulative asthma	Wheeze or whistling in the chest	Daytime attacks of breathlessness	Nocturnal attacks of breathlessness	Furry pet or pollen allergy	Respiratory infection
Age	0.52 (0.23–1.20)	1.29 (0.90–1.85)	1.15 (0.94–1.42)	2.09 (1.11–3.92)**	1.09 (0.67–1.78)	0.78 (0.64–0.95)*
Sex (boy = 0, girl = 1)	0.30 (0.09–0.97)*	0.76 (0.48–1.19)	1.65 (1.27–2.14)#	1.17 (0.54–2.55)	0.94 (0.51–1.71)	1.18 (0.93–1.51)
Parental asthma or allergy	2.67 (0.69–10.4)	2.66 (1.49–4.75)#	1.89 (1.26–2.83)#	0.96 (0.28–3.34)	1.81 (0.81–4.02)	0.98 (0.65–1.46)
Home environmental factors						
New painting	2.10 (0.54–8.18)	0.99 (0.52–1.87)	1.43 (0.97–2.11)	1.96 (0.76–5.06)	0.66 (0.26–1.71)	1.23 (0.84–1.80)
New floor	1.76 (0.31–10.1)	1.67 (0.77–3.60)	0.61 (0.35–1.04)	1.31 (0.39–4.38)	1.84 (0.66–5.14)	0.79 (0.47–1.31)
New furniture	0.83 (0.27–2.55)	1.76 (1.10–2.81)**	1.31 (1.00–1.72)**	1.24 (0.55–2.82)	1.47 (0.78–2.75)	1.03 (0.79–1.33)
ETS						
Never	1	1	1	1	1	1
1–3 times/month	1.05 (0.24–4.63)	1.94 (0.81–4.62)	1.23 (0.81–1.87)	1.59 (0.38–6.57)	0.67 (0.24–1.89)	1.90 (1.28–2.83)**
1–4 times/week	2.28 (0.54–9.52)	3.55 (1.51–8.39)**	1.61 (1.03–2.52)**	2.26 (0.54–9.43)	0.53 (0.16–1.82)	1.83 (1.19–2.82)**
Daily	0.16 (0.02–1.62)	2.29 (1.00–5.21)*	1.23 (0.83–1.83)	1.63 (0.42–6.39)	1.07 (0.44–2.61)	1.65 (1.13–2.42)**
Indoor air pollutants						
SO_2_	0.90 (0.24–3.42)	1.55 (1.06–2.27)**	1.16 (0.90–1.50)	1.06 (0.46–2.44)	1.11 (0.67–1.87)	0.89 (0.68–1.15)
NO_2_	3.20 (0.53–19.1)	0.71 (0.45–1.12)	0.77 (0.55–1.09)	1.46 (0.46–4.58)	0.94 (0.47–1.87)	1.08 (0.76–1.52)
O_3_	0.66 (0.15–2.89)	0.79 (0.42–1.51)	1.22 (0.82–1.81)	2.72 (1.03–7.18)**	0.95 (0.43–2.11)	0.98 (0.66–1.47)
CH_2_O^b^	1.11 (0.55–2.23)	1.11 (0.87–1.41)	0.93 (0.78–1.10)	1.92 (1.24–2.97)**	1.09 (0.79–1.51)	1.05 (0.88–1.24)
Outdoor air pollutants						
SO_2_	1.04 (0.53–2.07)	0.78 (0.59–1.03)	0.87 (0.73–1.03)	1.14 (0.71–1.82)	0.96 (0.67–1.37)	1.04 (0.88–1.24)
NO_2_	0.27 (0.04–1.63)	2.21 (0.99–4.98)	1.44 (0.88–2.37)	0.31 (0.08–1.20)	1.34 (0.48–3.75)	0.79 (0.48–1.29)
O_3_	4.24 (0.04–448)	0.12 (0.01–1.24)	0.31 (0.08–1.18)	17.9 (0.46–693)	0.44 (0.03–7.43)	1.28 (0.35–4.76)
CH_2_O^b^	4.61 (1.09–19.5)*	1.32 (0.86–2.04)	1.29 (0.99–1.68)	2.03 (0.91–4.54)	1.05 (0.60–1.85)	0.94 (0.72–1.23)

CH_2_O, formaldehyde.

a Three-level hierarchical logistic model (school-class-student) was applied with mutual adjustment with all factors (personal, home exposure, indoor and outdoor air pollutants) included in the model simultaneously. ORs for air pollutants both indoor and outdoor refer to a step change of 100, 10, 10, and 1 μg/m^3^ for SO_2_, NO_2_, O_3_, and formaldehyde, respectively. Air pollutants data with available measurements were applied.

b For the one school with missing formaldehyde measurement, the average value of available measurements of the other schools was applied.

* *p* < 0.05.

** *p* < 0.01.

# *p* < 0.001.
